# Varicella-Zoster Meningitis With Hypoglycorrhachia in an Immunocompetent Patient Presenting With Disseminated Varicella-Zoster Infection

**DOI:** 10.7759/cureus.8539

**Published:** 2020-06-09

**Authors:** Raman J Sohal, Sandeep Sohal, Tanya George, Tarvinder Gilotra

**Affiliations:** 1 Internal Medicine, State University of New York (SUNY) Upstate Medical University, Syracuse, USA; 2 Internal Medicine, The Brooklyn Hospital Center, Brooklyn, USA; 3 Infectious Disease, State University of New York (SUNY) Upstate Medical University, Syracuse, USA

**Keywords:** aseptic meningitis, vzv, varicella zoster, meningitis, hypoglycorrhachia, lumbar puncture, pcr, acyclovir, confusion, headache

## Abstract

Varicella-zoster virus (VZV) infection is rarely reported in immunocompetent hosts. We report the case of a 40-year-old male who presented with altered mental status. One week prior, he was seen at his outpatient physician’s office for a rash along the lateral right thigh. Erythema of the right gluteal region was noted, but no vesicles were present. He was treated for shingles rash with acyclovir with improvement. After a period of initial improvement in the rash, the patient developed a persistent headache. Given his migraine history, he overlooked the headache. He then developed fever, followed by confusion and was brought to the ED for further evaluation. CT head was unremarkable. Lumbar puncture revealed aseptic meningitis. This case highlights the unusual presentation of disseminated VZV infection in an immunocompetent host. It stresses the importance of maintaining high suspicion for disseminated VZV infection despite the patient being immunocompetent.

## Introduction

The risk of a varicella-zoster virus (VZV) infection increases in the population over the age of 50 (which account for 20% of the cases between the age group 50-59). Forty percent of the documented cases occur over the age of 60 years. The risk rises with increasing age and female gender. Immunocompromised people are at high risk due to the weakened activity of T-cell mediated immunity. Groups at risk in this category include lymphoma/leukemia patients, HIV, or people with autoimmune disease on systemic immunosuppressive therapy with chemotherapy, biologics as tumor necrosis factor (TNF), Janus kinase inhibitors (JAK2 kinase) or corticosteroids [1,2]. There have been few case reports of dermatomal distribution of VZV rash with aseptic meningitis in immunocompetent patient [3-5]. VZV is an uncommonly considered cause of meningitis and is frequently overlooked. However, given the appropriate clinical situation, VZV should always remain as high as a differential. 

Disseminated VZV can take two forms: cutaneous and visceral. Cutaneous disseminated VZV infection occurs when there is a lack of a dermatomal distribution of the rash. In our patient, there was extensive vesicular rash involving the proximal right lower extremity and gluteal region. It is usually associated with 20 or greater lesions that cross dermatomal distributions. Visceral disseminated VZV can take many forms, including involvement of the nervous system resulting in aseptic meningitis.

Hypoglycorrhachia is defined as a cerebrospinal fluid (CSF) glucose level of <45 mg/dL or CSF/serum ratio <0.5 [6]. It can occur due to anaerobic metabolism by the increased number of leukocytes (and/or bacterial cells) drawn into the CSF from the inflammatory response. To account for the changes seen with systemic hyperglycemia, the CSF/serum glucose ratio was used and two levels should be drawn within an hour of each other [6,7]. The normal CSF glucose concentration should be about 60% of the serum glucose level regardless of concomitant hyperglycemia as equilibration occurs, and this ratio is maintained. When it is less than 40%, it is termed hypoglycorrhachia, and it is most commonly associated with bacterial meningitis. However, there have been extensive studies that have looked at the occurrence of hypoglycorrhachia in aseptic meningitis. VZV has been a recently established pathogen capable of causing this phenomenon [2,3,6].

## Case presentation

We report the case of a 40-year-old healthy male with a history of hypertension and migraine who presented to the ED with a persistent headache and fever for five days. Review of systems was negative for changes in vision, speech, focal weakness, or numbness of the extremities. He did have a rash on the right lateral thigh and gluteal region. He reported that he was undergoing treatment for shingles infection with some improvement in the vesicular rash. There was no skin involvement on the left side. He did not report any history of recent travel or sick contacts. The physical exam did not show focal neurological signs and skin findings limited to the above description.

CT head non-contrast showed no acute intracranial hemorrhage, territorial infarct, or mass effect. The confusion, headache, and fever necessitated a lumbar puncture, which was performed. CSF analysis revealed elevated white blood cell count with lymphocytic predominance (121 nucleated cells (lymphocytes 71%)), elevated red blood cells (RBC 5 (reference range <2); likely from traumatic tap), mildly elevated protein (106 mg/dL; reference range 15-45 mg/dL), and normal glucose level (51 mg/dL reference range (40-71 mg/dL). These findings were consistent with aseptic meningitis. Gram stain of the CSF was negative. There was no bacterial growth of the CSF culture. Polymerase chain reaction (PCR) for VZV was performed, which was positive. Despite the CSF glucose level being low-normal when compared to serum glucose at that time, the CSF/serum glucose ratio was 0.3, which is abnormal. The patient was treated with intravenous acyclovir 750 mg every eight hours for 21 days. 

Lyme serologies were checked on the patient on admission, and he was positive for IgM antibodies, which was followed up with a positive confirmatory blot (positive for p39, p41). The result was equivocal, so qualitative Lyme PCR was sent out. In the interim, he was also started on doxycycline 100 mg twice daily for 14 days for empiric. It was unlikely that the patient had two primary CSF infections as per the infectious disease consulting service, even though the Lyme could appear consistent with lymphocytic meningitis. The repeat Lyme serologies were negative for both IgM and IgG, and the Lyme DNA qualitative PCR was negative. A CD4 count was also checked as it is unusual for multidermatomal VZV to occur in immunocompetent patients. The absolute CD4 count was 1437 (reference range 430-1800 cells/uL) with a CD4/D8 ratio of 4.1 (reference range 0.8 to 3.9). HIV was checked, which was negative for the HIV-1 p24 antigen and HIV-1/HIV-2 antibodies.

The patient followed on discharge three weeks later in the outpatient infectious disease clinic. At that time, the patient still had complained of mild headaches. He also endorsed a history of a red circular, discrete rash on his right lower extremity prior to his recent hospitalization for aseptic meningitis. A vesicular rash persisted on his right lateral thigh, although there was improvement along the gluteal region. At this point, the acyclovir duration was extended for one week, and the patient transitioned from intravenous to oral acyclovir therapy at 1 g three times per day.

## Discussion

VZV is not normally known to reoccur in a healthy adult population. Its presentation can be suggestive of underlying immunodeficiencies, such as HIV, malignancy, leukemia/lymphoma. This case is important for several reasons. Given the patient’s immunocompetent status, the probability of aseptic VZV meningitis was low. Secondly, given the hypoglycorrhachia, these findings were most suggestive of a bacterial infection. Despite this, given that the patient’s disseminated VZV infection involving several dermatomes, suspicion for a viral meningitis was high and CSF PCR panel revealed positive VZV. Lymphocytic predominance is seen in both viral and Lyme meningitis, which did further complicate the picture [2]. Given the positive IgM titers with a positive confirmatory immunoblot, the possibility of the Lyme meningitis could not be excluded. Therefore, the patient was treated with doxycycline 100 mg for two weeks. Fortunately, the labs were repeated, and Lyme DNA qualitative PCR was done, which was negative. This case emphasizes that it is very unlikely that two primary CSF infections would occur concomitantly, and therefore, clinical suspicion is key to ensuring an appropriate diagnosis. The history taken from the patient may be skewed by recall bias, particularly in the setting of rash descriptions. In this case, the patient mentioned the characteristic bull’s-eye rash as the initial cutaneous findings of Lyme disease and the patient agreed to have had a rash of this description. We could safely assume based on the repeat Lyme serologies, which were negative and the Lyme DNA qualitative PCR, which was negative that the initial presence of IgM for p39 and p41 was a false positive finding.

This case highlights the importance of understanding that hypoglycorrhachia has a broad differential and that although it is most commonly associated with bacterial meningitis, it should not exclude other causes such as VZV in this case [6]. A retrospective study by Shrikanth et al., looked at 602 patients with meningitis, in which case hypoglycorrhachia was present 19% of the time and 15% of it was due to viral etiology [6]. This should raise awareness among the internists to suspect VZV meningitis in patients with altered mentation not only when clinically supported (i.e., rash), but also in the absence of typical findings [8,9]. Also, less commonly reported etiologies include TB, HSV, Coxsackie and the Mumps virus. When present, hypoglycorrhachia was more suggestive of infection in immunocompromised patients with a vesicular rash present. In our case, the patient did have a rash but was immunocompetent.

## Conclusions

In conclusion, this case shows the atypical presentation of VZV infection in an immunocompetent patient. Our case aims to increase awareness of VZV as a cause of aseptic meningitis despite hypoglycorrhachia which has been historically more commonly associated with bacterial etiology. Particularly in this setting, the clinical presentation of a disseminated VZV with altered mental status, headache, and fever should strongly suggest the possibility of a CSF VZV infection, and not be overlooked as something more common given his history of migraine headache. Through our case, we hope to increase awareness of VZV as a cause of aseptic meningitis that can occur even in immunocompetent patients so that early treatment can be initiated for the best outcomes, particularly when supported by a suggestive clinical picture.

## References

[1] Imani S, Palavra NC, Oboudiyat C, Ip J (2020). Varicella-zoster meningitis in an immunocompetent young man presenting with a painless erythematous rash. BMJ Case Rep CP.

[2] Dawadi S, Lamsal S, Shah B (2020). Herpes zoster infection presenting as aseptic meningitis and dermatomal rash in immunocompetent adult. Case Rep Inf Dis.

[3] Gnoni M, Zaheer K, Vasser M (2018). Varicella zoster aseptic meningitis: report of an atypical case in an immunocompetent patient treated with oral valacyclovir. IDCases.

[4] Kangath R, Lindeman T, Brust K (2013). Herpes zoster as a cause of viral meningitis in immunocompetent patients. BMJ Case Rep.

[5] Takeshima S, Shiga Y, Himeno T (2017). Clinical, epidemiological and etiological studies of adult aseptic meningitis: report of 11 cases with varicella zoster virus meningitis [Japanese]. Rinsho Shinkeigaku.

[6] Shrikanth V, Salazar L, Khoury N, Wootton S, Hasbun R (2015). Hypoglycorrhachia in adults with community-acquired meningitis: etiologies and prognostic significance. Int J Infect Dis.

[7] Viola G (2010). Extreme hypoglycorrhachia: not always bacterial meningitis. Nat Rev Neurol.

[8] Fadhel M, Campbell N, Patel S (2019). Varicella zoster meningitis with hypoglycorrhachia on cerebrospinal fluid (CSF) analysis in a young immunocompetent host without a rash. Am J Case Rep.

[9] Spernovasilis N, Milioni A, Gialamas I, Kokorakis E, Fanti G (2018). Varicella-Zoster Virus meningitis with hypoglycorrhachia in a young immunocompetent adult without rash: a case report and literature review. IDCases.

